# Rotational and translational motions in a homogeneously cooling granular gas

**DOI:** 10.1038/s41526-024-00420-5

**Published:** 2024-07-31

**Authors:** Torsten Trittel, Dmitry Puzyrev, Kirsten Harth, Ralf Stannarius

**Affiliations:** 1 Department of Engineering, Brandenburg University of Applied Sciences, Magdeburger Str. 50, Brandenburg an der Havel, 14770 Germany; 2 MARS, Otto von Guericke University Magdeburg, Universitätsplatz 2, Magdeburg, 39106 Germany; 3 Department MTRM, Medical Faculty, Otto von Guericke University Magdeburg, Universitätsplatz 2, Magdeburg, 39106 Germany; 4 Institute of Physics, Otto von Guericke University Magdeburg, Universitätsplatz 2, Magdeburg, 39106 Germany

**Keywords:** Condensed-matter physics, Structure of solids and liquids

## Abstract

A granular gas composed of monodisperse spherical particles was studied in microgravity experiments in a drop tower. Translations and rotations of the particles were extracted from optical video data. Equipartition is violated, the rotational degrees of freedom were excited only to roughly 2/3 of the translational ones. After stopping the mechanical excitation, we observed granular cooling of the ensemble for a period of three times the Haff time, where the kinetic energy dropped to about 5% of its initial value. The cooling rates of all observable degrees of freedom were comparable, and the ratio of rotational and translational kinetic energies fluctuated around a constant value. The distributions of translational and rotational velocity components showed slight but systematic deviations from Gaussians at the start of cooling.

## Introduction

Granular gases are multi-particle systems that have been in the focus of scientific interest for decades. They were studied extensively in numerical simulations, because of the relatively simple, quasi-instantaneous interactions by individual dissipative collision events. In absence of external forces such as gravitation, each particle preserves its linear and angular momentum and kinetic energy until it collides with other particles (or, container walls). Kinetic energy is then partially transformed into other forms of energy, primarily thermal energy, and on average, the translational and rotational velocities of the constituents decrease. This process is commonly referred to as granular cooling. Already in the 1980s, Peter Haff^1^ proposed a scaling law for the kinetic energy loss of a homogeneously cooling dense granular gas of frictionless spheres. He predicted a time dependence of the form

1 $${E}_{{{{\rm{kin}}}}}(t)=\frac{{E}_{0}}{{(1+t/{\tau }_{{{{\rm{H}}}}})}^{2}},$$

which leads to the scaling *E*_kin_ ∝ *t*^−2^ for times *t* ≫ *τ*_H_. *E*_0_ is the kinetic energy at *t* = 0. The Haff time *τ*_H_(*E*_0_) defines the time scale of the energy loss. It depends upon several system parameters like the restitution coefficient of the particles, the mean particle velocity and the average collision rate. Details of the collisions play the decisive role in the ensemble dynamics.

Analytical and numerical studies in the past decades (see, e.g.,^2–28^) produced results that are strongly dependent upon simplifying assumptions. Zippelius^10^ gives some overview of numerical and analytical approaches and lists problems and open questions. Most studies dealt with spherical grains, but ellipsoidal and rod-like particles have been studied as well^14,24,25^. Experiments are still scarce, most of them were done in quasi two-dimensional (2D) layers (e.g.,^29–38^). The lack of experimental data is the major obstacle for a comparison and assessment of analytical studies and numerical simulations. Equipartition of kinetic energy among the individual degrees of freedom was analyzed in some of these 2D studies^32,35,37–39^. Recently, quantitative 3D experiments on granular cooling have been reported for rods^40,41^, spheres^42^ and ellipsoids^43^.

Here, we will focus on the rotational motion of the particles. Harth et al. experimentally studied the energy partition in a cooling 3D granular gas of rodlike particles (aspect ratio ≈ 7)^40,44^ in microgravity (*μ*g) in the ZARM drop tower in Bremen. The rod shape allowed a quantitative evaluation of both rotations and translations. We recall, that in thermodynamics, the heat capacity of mono-atomic gases at ambient temperatures is roughly 3/2 *kT*, since only three degrees of freedom (DOF) are involved, and ≈5/2 *kT* in two-atomic gases where five DOF are equally excited. The explanation requires quantum mechanics. Because of the very low moments of inertia for rotations about certain axes, even the lowest energy states are not excited at room temperature. For granular gases, owing to the macroscopic scale and the dissipative character of collisions, the behavior is different: For rod-shaped particles (which can be regarded as an analogue of two-atomic gases), the kinetic energy is essentially contained in five DOF^40^, but the mean energy per DOF for rotations about the short rod axes was found to be ≈20% lower than that of translations. The third rotation, about the long axis, was considerably less excited, roughly one order of magnitude lower^40^. Note that rotations about the short axes can be excited by all rod collisions that are not central, while rotations about the long rod axis are only be excited by frictional contacts. Numerical and analytical studies of spheres^3,10^ predicted that rotational DOF in a cooling granular gas retain more energy than the translational ones.

The focus of the evaluation is laid on the energy partition in a cooling 3D granular gas of spheres, where no quantitative experimental measurements of rotational motions have been available so far, and neither tests of energy partition. We further explore the distribution functions of velocities and rotation rates, the collision statistics and the cooling dynamics. Experimental data include video footage of drop tower experiments performed 2021 within the ESA “Drop your thesis!” program by the *SmartDust* project team (F. Guse, A. Murath, P. Boße, M. Zenker under the supervision of one of the authors (R.S.)) (see Methods section).

## Results

**Fig. 1:**
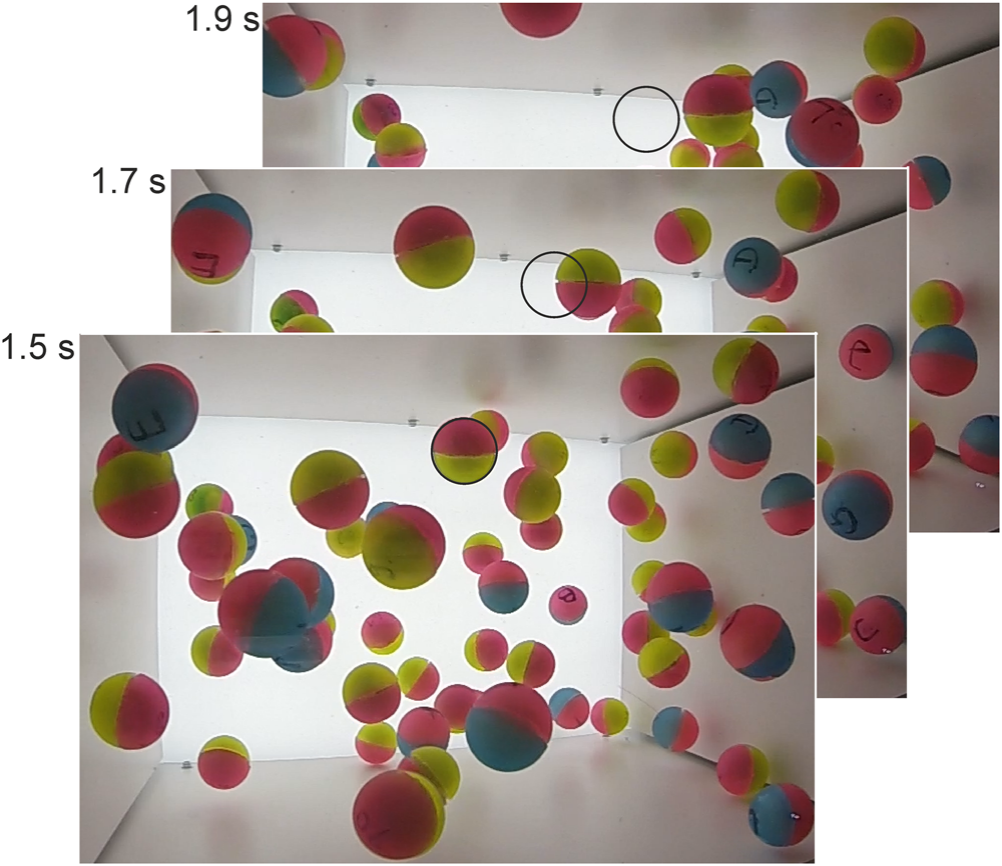
Camera snapshots of drop (I) with *N* = 57 balls at times 1.5 s, 1.7 s and 1.9 s after the excitation stopped in the *μg* phase. The marked particle performed a full rotation and moved by slightly more than 35 mm in the 0.4 s between the first and last image. The black circle indicates the particle position in the first image.

Rubber balls contained in a cuboid container were excited mechanically by two vibrated plates in the beginning of the experiment. After switching off the excitation in the microgravity phase, the dynamics of granular cooling was observed and analyzed. Data of two microgravity experiments in the ZARM drop tower in Bremen were used for the present evaluation. In drop (I), *N* = 57 soft rubber balls (see Methods section) were in the box, in drop (II), *N* = 88 balls. Mechanical excitation of the ensemble was initiated two seconds before the capsule drop. It was stopped 1 s after entry into weightlessness, so that a cooling period of approximately 3.7 s was available. Figure 1 shows examples of camera snapshots that were evaluated as described in the Methods section.

### Collision statistics

**Fig. 2:**
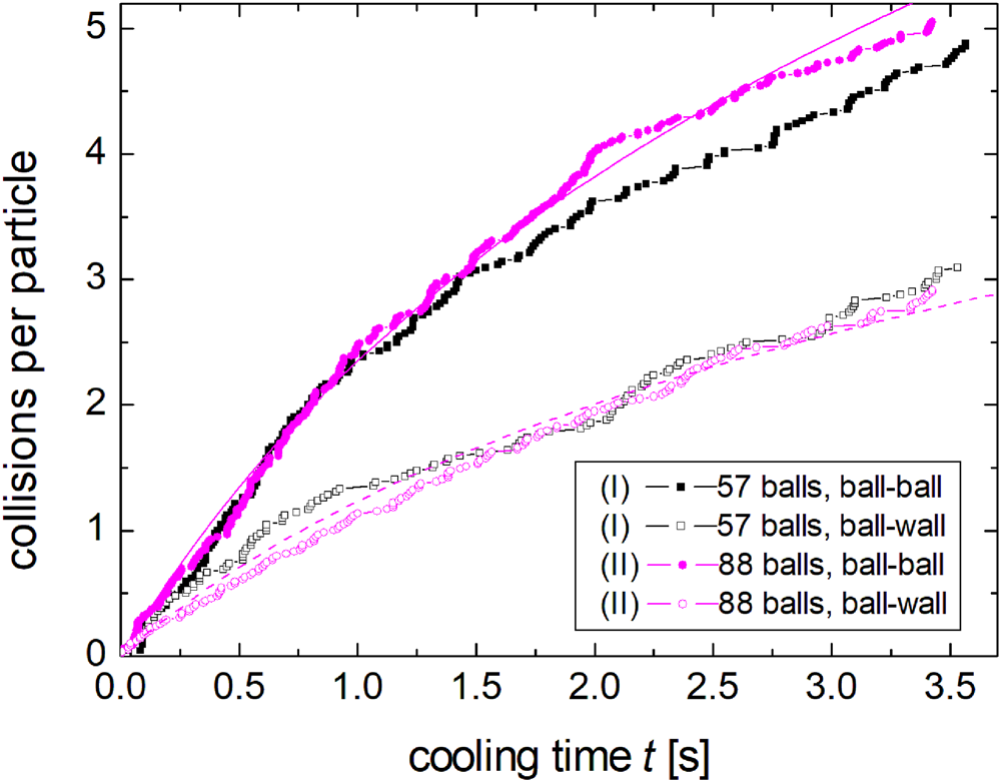
Cumulative plot of the average number of collisions per particle for both drops. The solid and dashed lines are logarithmic fits for drop (II), see text. Balls collide nearly twice as often with other balls as with the box.

The mean free path of the balls is $\lambda =1/(\sqrt{2}\pi n{d}^{2})$, with the particle number density *n* (number of particles *N* divided by the box volume *V*). In drop (I), *λ* ≈ 11 cm, and in drop (II), *λ* ≈ 7.5 cm. One can therefore expect that collisions with other balls dominate over wall contacts. Actually, counting the collisions revealed that slightly more contacts occurred between particles and container walls than between balls, but since the latter count twice, ball-ball collisions dominate the statistics for the individual particles (Fig. 2). The approximate ratio is 1.9 in drop (II), and about 10% less in drop (I). The average collision rate of a particle is given by $\bar{V}/\lambda$, where $\bar{V}$ is the mean particle velocity. The expected cumulative number of collisions during homogeneous cooling is^24,40,45^

2 $${N}_{{{{\rm{C}}}}}(t)={\xi }^{-1}{(1-{\varepsilon }^{2})}^{-1}\ln \left(1+t/{\tau }_{{{{\rm{H}}}}}\right),$$

where *t* = 0 at the start of cooling. Here, *ε* is the restitution coefficient, *ξ* is a dimensionless parameter related to the number of DOF of the system. Both ball–wall and ball–ball collisions follow such a logarithmic trend, and the total number of collisions can be well approximated setting the dimensionless parameter *ξ* ≈ 0.85. and the value of *τ*_*H*_ given below.

### Translational and rotational velocity distributions

**Fig. 3:**
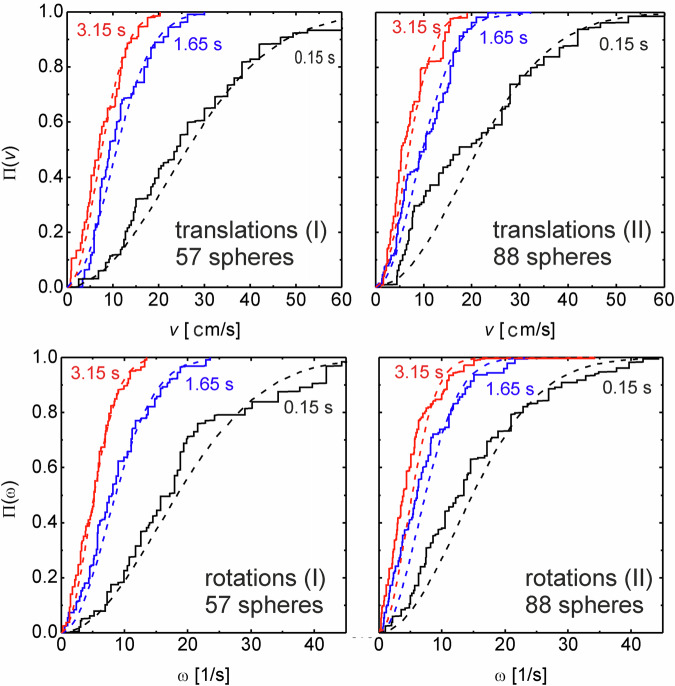
Cumulative probabilities of translational velocities (top row) and rotational velocities (bottom row) during cooling, see text. The curves correspond to intervals of 0.3 s centered around 0.15, 1.65 and 3.15 s, respectively. Dashed lines indicate integrated MB velocity distributions with the same mean squared velocities as the experiment.

Next, we analyze the velocity distribution of the transverse velocity component, perpendicular to the viewing direction. We present cumulative probabilities Π(*v*) that the transverse particle velocity ${v}^{{\prime} }$ is smaller than *v* and cumulative probabilities Π(*ω*) that the rotational velocity ${\omega }^{{\prime} }$ is lower than *ω*,

3 $$\Pi (v)=\int_{0}^{v}p({v}^{{\prime} })d{v}^{{\prime} },\quad \Pi (\omega )=\int_{0}^{\omega }p({\omega }^{{\prime} })d{\omega }^{{\prime} },$$

at different stages of cooling, where $p({v}^{{\prime} })$ is the distribution density of the transverse velocity ${v}^{{\prime} }$ and $p({\omega }^{{\prime} })$ is the analogue for ${\omega }^{{\prime} }$. As a compromise of satisfactory statistics and sufficiently narrow time ranges, we averaged the data over 0.3 s intervals. Figure 3 shows the results for intervals centered around times 0.15 s, 1.65 s and 3.15 s after the excitation was switched off. Note that each of these dynamic variables accounts for 2 DOF. A Maxwell-Boltzmann (MB) model in 2D would yield the distributions

4 $$p(v)=\frac{\pi v}{2{\bar{v}}^{2}}\cdot \exp \left(-\frac{\pi {v}^{2}}{4{\bar{v}}^{2}}\right),\quad \Pi (v)=1-\exp \left(-\frac{\pi {v}^{2}}{4{\bar{v}}^{2}}\right)$$

with the mean transverse velocity $\bar{v}(t)$, and $\overline{{v}^{2}}=\frac{4}{\pi }{\bar{v}}^{2}$. This model is shown in Fig. 3 with dashed lines, where the mean squared velocities $\overline{{v}^{2}}$ were taken from the experimental distributions. Note that under the assumption of the MB model and equivalence of all three translational DOF, the mean absolute (3D) velocity is $\bar{V}=\pi \bar{v}/2$.

Analogous equations were evaluated for the rotational motions ($v,\bar{v}$ replaced by $\omega ,\bar{\omega }$). The main result of this evaluation is that the MB model fits the distributions at least in reasonable approximation. At high velocities, the experimental cumulative probability curves in Fig. 3 are systematically below the MB model curves, particularly at the start of cooling. This reflects a smaller share of slower balls. Particles with high velocities and rotational velocities (hot particles) are systematically over-represented. The actual distributions are slightly broader than assumed in the MB model.

**Fig. 4:**
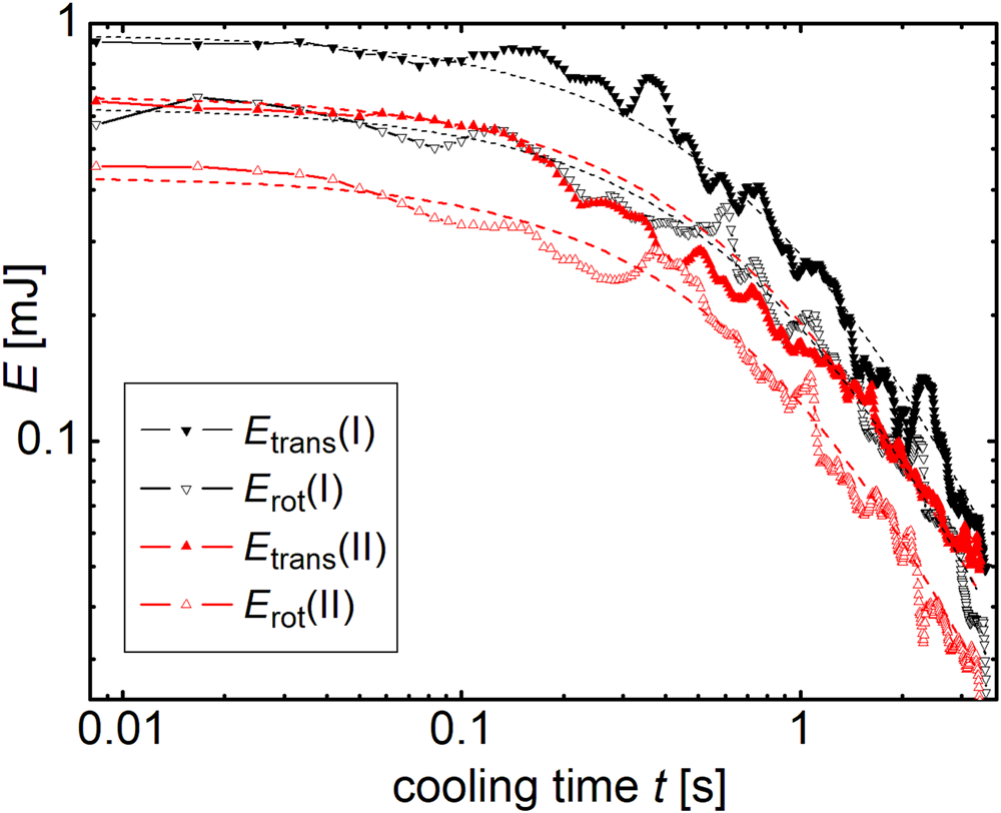
Kinetic energies per translational and rotational DOF for the two drop experiments. The dashed lines are fits with Eq. (1) and a Haff time *τ*_H_ of 1.2 s.

Table 1 lists the experimental results. The mean velocities of both experiments are roughly comparable, with initially about 20% lower mean values for experiment (II), the one with more particles. Figure 4 shows the time dependence of the kinetic energies per DOF in both experiments. The kinetic energy drops by more than one order of magnitude. Both experiments can be described with Haff’s Eq. (1) and *τ*_H_ = 1.2 ± 0.08 s. The collision statistics confirms that the differences between both experiments are marginal. One might expect faster cooling for system (II) with higher particle number density, but this is compensated here by the higher velocities in drop (I) at the end of the heating phase. The kinetic energies achieved with the same excitation parameters are significantly larger in drop (I). Initial energies per translational DOF, taken from the Haff fits in Fig. 4 were 0.94 mJ (I) and 0.67 mJ (II), respectively. Per rotational DOF, the energies after heating were 0.63 mJ (I) and 0.43 mJ (II), respectively.

**Table 1:** Parameters ${\overline{v}}$ and ${\overline{\omega}}$ at different stages of cooling

exp. no.	*N*	quantity	0.0…0.3 s	1.5…1.8 s	3.0…3.3 s
(I)	57	$\bar{v}$ [cm/s]	26.9	11.3	7.7
(II)	88	$\bar{v}$ [cm/s]	21.1	10.8	7.1
(I)	57	$\bar{\omega }$ [1/s]	18.8	8.8	5.38
(II)	88	$\bar{\omega }$ [1/s]	14.8	7.10	4.85

All data are averages over 0.3 s periods. The statistical accuracy of the values is 10%.

The fluctuations in the energy graphs mainly arise from the evaluation statistics. Since not all of the balls can be included in the momentary velocity or rotation rate statistics because of overlapping, obscuring and collisions, part of the total energy is contained in the ‘hidden’ particles and the distribution of kinetic energies between evaluated and hidden balls fluctuates statistically by about 10%. This does not affect the overall cooling trend.

### Energy partition

**Fig. 5:**
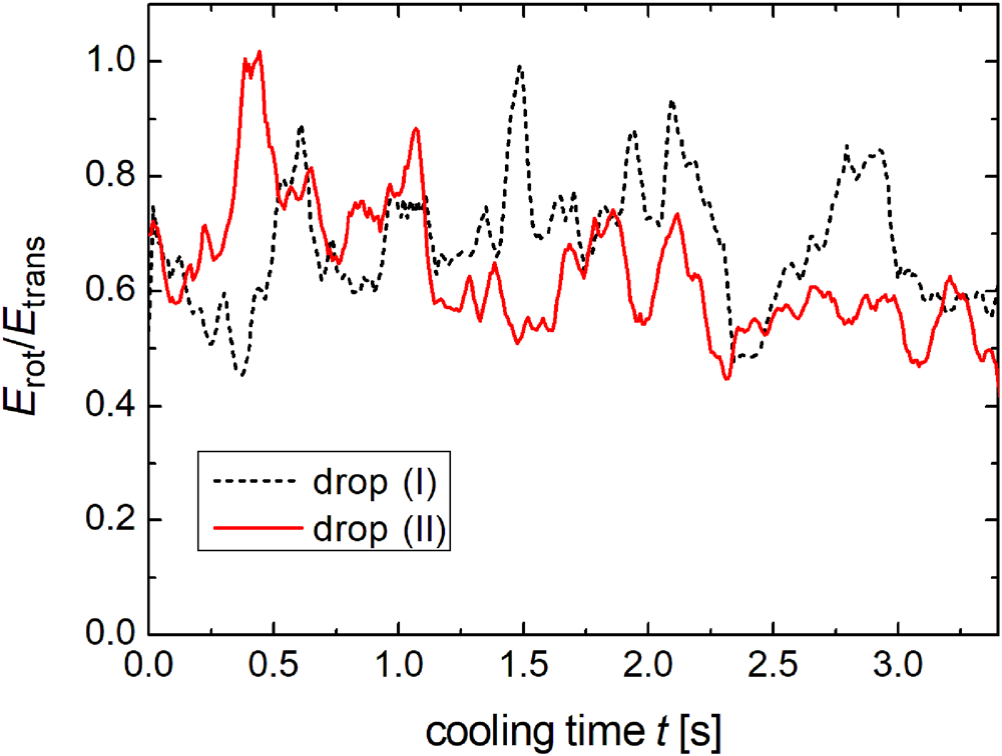
Ratio of energies of the rotational and translational DOF for both experiments. The values fluctuate, but do not show a significant trend with progressing cooling time or filling fraction.

Figure 5 shows the evolution of the ratio of kinetic energies in the rotational and translational DOF. The smaller, fast fluctuations ( < 100 ms) are within the statistical uncertainty, but the larger, slow fluctuations reflect actual system dynamics. The statistical error is about ±15%. The main error source comes from the evaluation procedure: Short trajectories between two collisions cannot be evaluated. Some particles are temporarily obscured by others, thus only about 70% of all spheres enter the statistics at each instant. We assume that these objects follow the same statistics as the evaluated ones, due to the fact that the instantaneous velocity and rotation rate does not affect the momentary visibility of the spheres. Energies may not only be transferred among the DOF plotted in Fig. 4, but also to the non-visible DOF or to particles temporarily not included in the evaluation. This may even cause apparent small increases in the sum (*E*_rot_ + *E*_trans_) of Fig. 4. Apart from these fluctuations, there is no significant trend in the ratio of *E*_rot_/*E*_trans_ during the cooling process for either of the experiments. This is consistent with the similar Haff times.

**Fig. 6:**
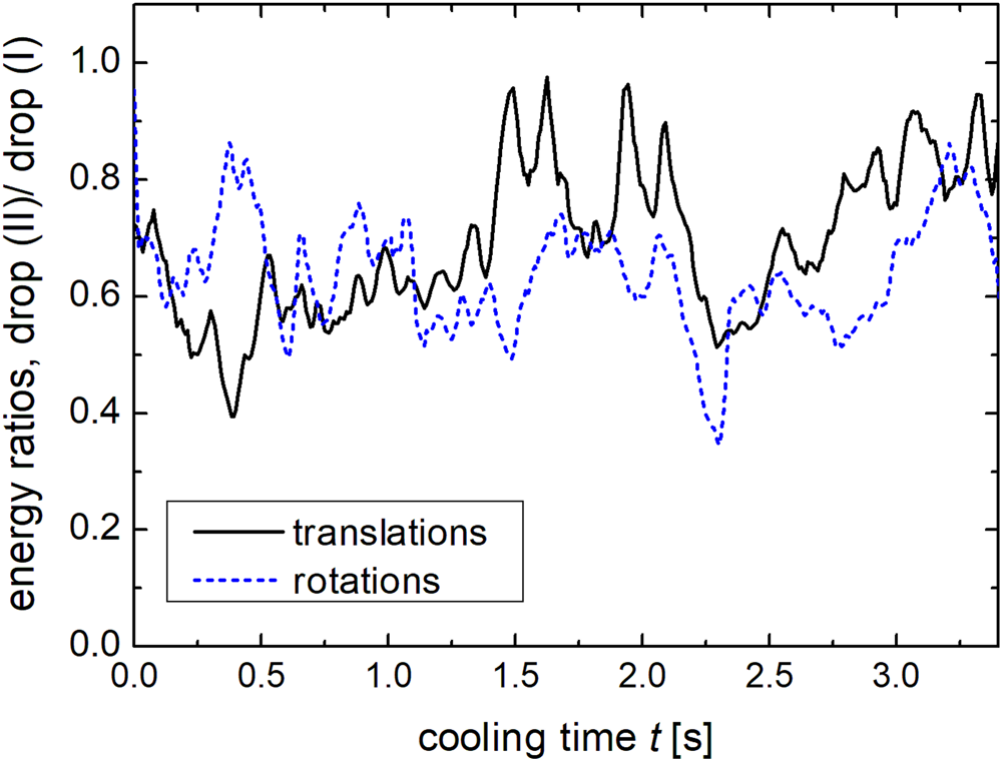
Comparison of the mean kinetic energies for the two drop experiments. Ratio of the mean kinetic rotational and translational energies per particle for 88 particles (drop II) versus 57 particles (drop I) during cooling.

In the same way, Fig. 6 compares the ratio of kinetic energies of both drops. As expected, there are considerable fluctuations but no clear trend. The total kinetic energy per particle in the denser system (II) is on average 20–40% lower than in the more diluted system (I). The reason for that is that the initial energy after excitation is lower in the denser configuration because the system approaches a state where the energy lost in collisions and the energy entry by the vibrating walls compensate each other on average. Then, this initial ratio is basically maintained because of the comparable Haff times.

## Discussion

Our study has provided the first quantitative comparison of rotational and translational motions of spherical particles in a cooling granular gas experiment. The rotational DOF are excited to about 60–80% of the translational ones. All DOF have similar cooling behavior, so that the ratio of kinetic energies fluctuates around a constant value during cooling, without a visible trend. On average, each particle collides nearly twice as often with other particles as with walls. Since bouncing of our soft particles at the hard wall can be considered as a collisions with a similar virtual mirror particle, the presence of boundaries will not distort our results dramatically. No indications of a developing spatial inhomogenity were found in the optical data.

Comparison with existing theoretical studies confirms some of their predictions qualitatively. Cooling of rough spheres was described by Huthmann and Zippelius^4^. In agreement with their analytical results, rotational and translational energies finally decay with a constant ratio. In our experiment, this ratio is already established at the beginning of the cooling phase, whereas the theoretical study started with purely translational motion. A striking difference between experiment and simulation is that we find a ratio *R* = *E*_rot_/*E*_trans_ smaller than unity, while it is well above 1 in their model. Zippelius^10^ considered an initial state with *R*(0) = 0. The crossover *R* = 1 occurred after a period of the order of 1000 *τ*_H_. The ratio further rose above 3, and a final constant ratio was not even reached after more than 10^5^*τ*_H_. The latter predictions seem to differ from our results. Luding et al.^3^ also considered a system starting with *R* = 0. They described the energy loss as first linear, then as quadratic decay, but the linear approximation may only be useful for short times *t* ≪ *τ*_H_. The main predictions of that paper refer to an initial period much shorter than *τ*_H_ (few dozen milliseconds). At later times, a constant *R* is found, albeit with a value larger than unity in contrast to our measurements. Further systematic experiments with particles of different collision properties, including normal and effective transverse restitution coefficients, are needed to clarify these discrepancies.

Future theoretical and numerical studies will benefit from detailed experimental data. The latter provide benchmarks to validate numerical approaches and to improve a realistic description of dilute multi-particle ensembles. A substantial progress in the experimental characterization may be the usage of smart particles with integrated boards that record rotational motions and accelerations of the translational motion^46^.

## Methods

### Setup and materials

The setup for the experiments in microgravity in the ZARM drop tower in Bremen was designed and constructed for an ESA “Drop your thesis” student project under supervision by one of the authors (R. S.). The original purpose of that experiment was to collect data from intelligent sensors embedded in selected particles. This part was not successful for technical reasons, but the collected optical video data provide valuable information to determine translations and rotations of the spheres for a statistical analysis.

The setup consists of a 30 × 30 × 40 cm^3^ cuboid box. Two opposite walls of the box are attached to loudspeakers so that they could be vibrated with a sine frequency of 20 Hz and amplitudes up to 10 mm. These two walls are made of painted aluminum. The other walls are made from perspex glass. The front plate is transparent. The entire ensemble was observed with two video cameras *GoPro Hero 3 Ribcage* with c-mount objective lenses. The image resolution is 1280 × 720 pixel^2^ at a frame rate of 120 fps. From the stereoscopic views, a 3D reconstruction would be possible, but was not intended here.

The particles are elastic rubber balls (*Flummies*), acquired from a commercial supplier. They have a diameter of *d* = 35 mm and a mass of *m* = 22.4 g. The elastic modulus is 1.7 MPa, and the restitution coefficient is *ε* = 0.90, independent of the impact speed in the velocity range relevant here. The friction coefficient *μ* is approximately one. All balls consist of two differently colored hemispheres. This allows to retrieve information on two rotational degrees of freedom. The third one, rotations about the axis perpendicular to the equator, is not accessible. For symmetry reasons, we presume that the rotations about all three axes are statistically equivalent.

The ZARM drop tower is equipped with a catapult that can provide up to 9.2 s microgravity time. Since the catapult was not available for the campaign, only drops could be realized, which reduced the *μg* time to 4.7 s. A total of six drop experiments were performed. The extraction of particle velocities and rotation rates is the most time-consuming phase of the evaluation. Thus, two of the experiments were evaluated in the present study. The remaining four did not provide relevant additional information. They confirm the results presented here. The number of balls in the individual drop experiments ranged from 55 to 110. The two drops evaluated here were performed with 57 particles (volume fraction *ϕ* = 0.0355) and 88 particles (*ϕ* = 0.0549), respectively.

### Data acquisition

Footage of one of the cameras was selected for evaluation of the particle dynamics. The material of the second camera was not used for the statistical analysis, since it doubles the evaluation efforts without providing much additional information. By random sampling, we checked consistency with the first camera data.

We employ the following approach to obtain a statistics of the translations of the spheres in the viewing plane: A particle is selected and marked in a given frame and tracked in subsequent frames (Fig. 1). We determine the number of frames passed until the particle has moved by one sphere diameter without colliding. This is when the backward edge of the sphere in the direction of motion has reached the initial position of the forward edge. Thus, we avoid the necessity to correct perspective distortions of the images. If the particle moves exactly in the viewing plane, this approach gives the exact in-plane velocity. If it has a velocity component along the viewing direction, we detect only the displacement normal to that direction. The projected path is the average of the apparent sphere diameters in the first and the last frame of the interval in good approximation. If the particle encounters a collision before traveling one diameter, the path until the collision occurs is counted and evaluated. A new interval starts after the collision. When the motion is very fast, we can interpolate between frame numbers. In the beginning, particles at the most probable speed need about 1/7 s (≈17 frames) to travel sideways by one diameter. Thus, even without interpolation of frames, the accuracy of the translational velocity data is better than 5%, and it improves as cooling progresses and slows speeds down.

Rotation rates of the spheres are determined in the following way: The image of a selected sphere in a given frame is taken as reference. Then, we determine the frame where this sphere has the same appearance again (Fig. 1), after having performed a full rotation about an axis in its equatorial plane. Again, we consider only periods, where the particle does not collide. The corresponding time lag yields the angular velocity *ω* = *ω*_⊥_. This quantity represents two rotational DOF. Rotations about the axis perpendicular to the particle equator are not detected. The average number of frames for a full rotation in the beginning is of the order of 20 (≈1/6 s). Rotations by 180^∘^ are also suitable to determine *ω*: After half a rotation, the two hemispheres are exchanged.

## Data Availability

Raw data are provided by the corresponding author on request.

## References

[1] Haff, P. K. Grain flow as a fluid-mechanical phenomenon. *J. Fluid Mech.* **134**, 401 (1983). 10.1017/S0022112083003419

[2] Goldhirsch, I. & Zanetti, G. Clustering instability in dissipative gases. *Phys. Rev. Lett.* **70**, 1619 (1993). 10053341 10.1103/PhysRevLett.70.1619

[3] Luding, S., Huthmann, M., McNamara, S. & Zippelius, A. Homogeneous cooling of rough, dissipative particles: theory and simulations. *Phys. Rev. E* **58**, 3416 (1998). 10.1103/PhysRevE.58.3416

[4] Huthmann, M. & Zippelius, A. Dynamics of inelastically colliding rough spheres: relaxation of translational and rotational energy. *Phys. Rev. E* **56**, R6275 (1997). 10.1103/PhysRevE.56.R6275

[5] Huthmann, M., Aspelmeier, T. & Zippelius, A. Granular cooling of hard needles. *Phys. Rev. E* **60**, 654 (1999). 10.1103/PhysRevE.60.654 11969806

[6] Cafiero, R., Luding, S. & Herrmann, H. J. Two-dimensional granular gas of inelastic spheres with multiplicative driving. *Phys. Rev. Lett.* **84**, 6014 (2000). 10991112 10.1103/PhysRevLett.84.6014

[7] Cafiero, R., Luding, S. & Herrmann, H. J. Rotationally driven gas of inelastic rough spheres. *Europhys. Lett.* **60**, 854 (2002). 10.1209/epl/i2002-00295-7

[8] Herbst, O., Cafiero, R., Zippelius, A., Herrmann, H. J. & Luding, S. A driven two-dimensional granular gas with Coulomb friction. *Phys. Fluids* **17**, 107102 (2005). 10.1063/1.2049277

[9] Evesque, P., Palencia, F., Lecoutre-Chabot, C., Beysens, D. & Garrabos, Y. Granular gas in weightlessness: the limit case of very low densities of non interacting spheres. *Microgravity Sci. Technol.* **XVI-I**, 280 (2005). 10.1007/BF02945991

[10] Zippelius, A. Granular gases. *Phys. A* **369**, 143 (2006). 10.1016/j.physa.2006.04.012

[11] Brilliantov, N. V., Pöschel, T., Kranz, W. T. & Zippelius, A. Translations and rotations are correlated in granular gases. *Phys. Rev. Lett.* **98**, 128001 (2007). 17501156 10.1103/PhysRevLett.98.128001

[12] Ben-Naim, E. & Zippelius, A. Singular energy distributions in driven and undriven granular media. *J. Stat. Phys.* **129**, 677 (2007). 10.1007/s10955-007-9411-0

[13] Polito, A., Filho, T. R. & Figueiredo, A. On the velocity distributions of granular gases. *Phys. Lett. A* **374**, 13 (2009). 10.1016/j.physleta.2009.10.025

[14] Kanzaki, T., Hidalgo, R. C., Maza, D. & Pagonabarraga, I. Cooling dynamics of a granular gas of elongated particles. *J. Stat. Mech.* **6**, P06020 (2010).

[15] Daniels, L. J. & Durian, D. J. Propagating waves in a monolayer of gas-fluidized rods. *Phys. Rev. E* **83**, 061304 (2011). 10.1103/PhysRevE.83.061304 21797355

[16] Bodrova, A. & Brilliantov, N. Self-diffusion in granular gases: an impact of particles’ roughness. *Granul. Matt.* **14**, 85 (2012). 10.1007/s10035-012-0319-2

[17] Bodrova, A., Levchenko, D. & Brilliantov, N. Universality of temperature distribution in granular gas mixtures with a steep particle size distribution. *EPL* **106**, 14001 (2014). 10.1209/0295-5075/106/14001

[18] Chen, Y., Evesque, P. & Hou, M. Asymmetric local velocity distribution in a driven granular gas. *Eng. Comput.* **32**, 1066 (2015). 10.1108/EC-04-2014-0089

[19] Vega Reyes, F., Lasanta, A., Santos, A. & Garzo, V. Energy nonequipartition in gas mixtures of inelastic rough hard spheres: the tracer limit. *Phys. Rev. E* **96**, 052901 (2017). 29347772 10.1103/PhysRevE.96.052901

[20] Brilliantov, N. V., Formella, A. & Poeschel, T. Increasing temperature of cooling granular gases. *Nat. Comm.* **9**, 797 (2018). 10.1038/s41467-017-02803-7 PMC5824832 29476073

[21] Santos, A. Interplay between polydispersity, inelasticity, and roughness in the freely cooling regime of hard-disk granular gases. *Phys. Rev. E* **98**, 012904 (2018). 30110735 10.1103/PhysRevE.98.012904

[22] Lasanta, A., Vega Reyes, F., Garzo, V. & Santos, A. Intruders in disguise: mimicry effect in granular gases. *Phys. Fluids* **31**, 063306 (2019). 10.1063/1.5097398

[23] Bodrova, A. S., Osinsky, A. & Brilliantov, N. V. Temperature distribution in driven granular mixtures does not depend on mechanism of energy dissipation. *Sci. Rep.* **10**, 1 (2020). 31959873 10.1038/s41598-020-57420-0 PMC6971070

[24] Villemot, F. & Talbot, J. Homogeneous cooling of hard ellipsoids. *Granul. Matter* **14**, 91 (2012). 10.1007/s10035-012-0322-7

[25] Rubio-Largo, S. M., Alonso-Marroquin, F., Weinhart, T., Luding, S. & Hidalgo, R. C. Homogeneous cooling state of frictionless rod particles. *Physica A*. **433**, 477 (2016). 10.1016/j.physa.2015.09.046

[26] Megías, A. & Santos, A. Hydrodynamics of granular gases of inelastic and rough hard disks or spheres. I. Transport coefficients. *Phys. Rev. E* **104**, 034901 (2021). 34654090 10.1103/PhysRevE.104.034901

[27] Megías, A. & Santos, A. Hydrodynamics of granular gases of inelastic and rough hard disks or spheres. II. Stability analysis. *Phys. Rev. E* **104**, 034902 (2021). 34654064 10.1103/PhysRevE.104.034902

[28] Kremer, G. M. & Santos, A. Granular gas of inelastic and rough Maxwell particles. *J. Stat. Phys.* **189**, 23 (2022). 10.1007/s10955-022-02984-6

[29] Olafsen, J. S. & Urbach, J. S. Clustering, order, and collapse in a driven granular monolayer. *Phys. Rev. Lett.* **81**, 4369 (1998). 10.1103/PhysRevLett.81.4369

[30] Olafsen, J. S. & Urbach, J. S. Velocity distributions and density fluctuations in a granular gas. *Phys. Rev. E* **60**, R2468 (1999). 10.1103/PhysRevE.60.R2468 11970178

[31] Kudrolli, A., Wolpert, M. & Gollub, J. P. Cluster formation due to collisions in granular material. *Phys. Rev. Lett.* **78**, 1383 (1997). 10.1103/PhysRevLett.78.1383

[32] Feitosa, K. & Menon, N. Breakdown of energy equipartition in a 2D binary vibrated granular gas. *Phys. Rev. Lett.* **88**, 198301 (2002). 12005668 10.1103/PhysRevLett.88.198301

[33] Maaß, C. C., Isert, N., Maret, G. & Aegerter, C. M. Experimental investigation of the freely cooling granular gas. *Phys. Rev. Lett.* **100**, 248001 (2008). 18643629 10.1103/PhysRevLett.100.248001

[34] Tatsumi, S., Murayama, Y., Hayakawa, H. & Sano, M. Experimental study on the kinetics of granular gases under microgravity. *J. Fluid Mech.* **641**, 521 (2009). 10.1017/S002211200999231X

[35] Grasselli, Y., Bossis, G. & Goutallier, G. Velocity-dependent restitution coefficient and granular cooling in microgravity. *Europhys. Lett.* **86**, 60007 (2009). 10.1209/0295-5075/86/60007

[36] Burton, J. C., Lu, P. Y. & Nagel, S. R. Energy loss at propagating jamming fronts in granular gas clusters. *Phys. Rev. Lett.* **111**, 188001 (2013). 24237564 10.1103/PhysRevLett.111.188001

[37] Nichol, K. & Daniels, K. E. Equipartition of rotational and translational energy in a dense granular gas. *Phys. Rev. Lett.* **108**, 018001 (2012). 22304293 10.1103/PhysRevLett.108.018001

[38] Grasselli, Y., Bossis, G. & Morini, R. Translational and rotational temperatures of a 2D vibrated granular gas in microgravity. *Eur. Phys. J. E* **38**, 8 (2015). 10.1140/epje/i2015-15008-5 25681008

[39] Daniels, L. J., Park, Y., Lubensky, T. C. & Durian, D. J. Dynamics of gas-fluidized granular rods. *Phys. Rev. E* **79**, 041301 (2009). 10.1103/PhysRevE.79.041301 19518218

[40] Harth, K., Trittel, T., Wegner, S. & Stannarius, R. Free cooling of a granular gas of rodlike particles in microgravity. *Phys. Rev. Lett.* **120**, 214301 (2018). 29883145 10.1103/PhysRevLett.120.214301

[41] Puzyrev, D., Harth, K., Trittel, T. & Stannarius, R. Cooling of a granular gas mixture in microgravity. *npj Microgravity* **10**, 36 (2024). 38519479 10.1038/s41526-024-00369-5 PMC10959983

[42] Yu, P., Schroeter, M. & Sperl, M. Velocity distribution of a homogeneously cooling granular gas. *Phys. Rev. Lett.* **124**, 208007 (2020). 32501095 10.1103/PhysRevLett.124.208007

[43] Pitikaris, S., Bartz, P., Yu, P., Cristoforetti, S. & Sperl, M. Granular cooling of ellipsoidal particles in microgravity. *NPJ Microgravity* **8**, 11 (2022). 35444243 10.1038/s41526-022-00196-6 PMC9021203

[44] Harth, K. et al. Granular gases of rod-shaped grains in microgravity. *Phys. Rev. Lett.* **110**, 144102 (2013). 25166993 10.1103/PhysRevLett.110.144102

[45] Costantini, G., Marconi, U. M. B., Kalibaeva, G. & Cicotti, G. The inelastic hard dimer gas: a nonspherical model for granular matter. *J. Chem. Phys.* **122**, 164505 (2005). 15945691 10.1063/1.1884999

[46] Trittel, T., Puzyrev, D. & Stannarius, R. Platonic solids bouncing on a vibrating plate. *Phys. Rev. E* **109**, 034903 (2024). 38632736 10.1103/PhysRevE.109.034903

